# Enhancing aphid detection framework based on ORB and convolutional neural networks

**DOI:** 10.1038/s41598-020-75721-2

**Published:** 2020-10-29

**Authors:** Haoran Pei, Kui Liu, Xiaojing Zhao, Ali Abdullah Yahya

**Affiliations:** 1 School of Computer and Information, Anqing Normal University, Anqing, 246133 Anhui China; 2 The Key Lab of Intelligent Perception and Computing of Anhui Province, Anqing, 246133 Anhui China

**Keywords:** Computer science, Information technology

## Abstract

Methods to detect directly aphids based on convolutional neural networks (CNNs) are unsatisfactory because aphids are small and usually are specially distributed. To enhance aphid detection efficiency, a framework based on oriented FAST and rotated BRIEF (ORB) and CNNs (EADF) is proposed by us to detect aphids in images. Firstly, the key point is to find regions of aphids. Points generated by the ORB algorithm are processed by us to generate suspected aphid areas. Regions are fed into convolutional networks to train the model. Finally, images are detected in blocks with the trained model. In addition, in order to solve the situation that the coordinates are not uniform after the image is segmented, we use a coordinate mapping method to unify the coordinates. We compare current mainstream target detection methods. Experiments indicate that our method has higher accuracy than state-of-the-art two-stage methods that the AP value of RetinaNet with EADF is 0.385 higher than RetinaNet without it and the Cascade-RCNN with EADF is more than without it by 43.3% on value of AP, which demonstrates its competency.

## Introduction

Aphids are one of the most destructive pests on the planet. They are harmful to crops and can lead to reduced yields. In addition, aphids are small and numerous. To protect crops and increase yields, it is necessary to correctly identify aphids and take corresponding measures. Manual identification takes considerable time and effort, and its accuracy is sometimes less than desired. There are many novel methods to improve the detection speed and accuracy. Among traditional detection methods^1^, proposed to use the histograms of oriented gradient descriptors for pedestrian detection, which has good geometric and optical invariance. The image is divided into many subgraphs, called cells, and each cell calculates a gradient direction histogram. Cells in a block are then normalized. Finally, combining the classifier to identify. A model was proposed based on a pictorial framework^2^. New local and semi-local features play an important role in target detection. Unlike exhaustive types of algorithms^3^, combined exhaustive search and segmentation. The whole picture is divided into several small areas, which are merged into larger areas based on similarity of color, texture, and area, and fill similarity between the areas. These merged areas are also called region proposals.

With the rapid development of computer vision, many neural network-based methods are gaining wide use. LeNet-5 is an efficient convolutional neural network for handwritten character recognition. It has been used in^4^ and achieved very good results. Later, Alex krizhevsky proposed AlexNet in^5^. AlexNet structure is similar to LeNet on the whole, but different in details. AlexNet uses relu as the activation function instead of sigmoid and uses dropout to prevent over fitting. Compared with AlexNet, which uses 11*11 and 5*5 convolution kernels, VGG^6^ uses 3 * 3 convolution kernels to more easily capture the changes of image feature details. The one-stage method, with a convolutional neural network (CNN) framework, has a capacity to directly mark the position of the target with considerable speed. The core idea of^7^ is to solve object detection as a regression task. YOLO has a simple framework and can simultaneously predict the position and category of the bounding box. It is worth noting that YOLO has considerable detection speed. Unlike YOLO, the SSD^8^ algorithm directly uses convolution in the last layer to extract the results, and it uses feature maps of different sizes to detect targets of diverse sizes. As is well known, one-stage detectors widely use anchor boxes to obtain better detection results. However, the method proposed in^9^ replaces anchor boxes with regions generated by key points calculated by convolutional networks. Corner pooling, as a novel component, is used by CornerNet to locate better corner points of a box. The accuracy of one-stage detectors is affected by the class imbalance. The problem of low accuracy has been addressed by a new loss function and a one-stage framework called RetinaNet^10^. Two-stage detectors have achieved higher accuracy. Based on candidate regions calculated by selective search^3^, CNNs have been used to identify objects^11^. R-CNN is the first algorithm to successfully apply deep learning to object detection. Subsequently, based on R-CNN^11^, fast RCNN^12^ and faster RCNN^13^ were proposed. Ref.^14^ was mainly aimed at IoU threshold selection in detection problems. To ensure a high-quality proposal without reducing training samples, a method was proposed that uses the output of one stage to train the next stage. Bin Xue,Ningning Tong and Xin Xu propose a method called DIOD^15^, which is based on full convolution region candidate network and fast semi-supervision of deep convolutional neural networks. Qian Yan et al.^16^utilises the deep convolutional neural network to identify apple leaf disease. With the development of CNNs, the number of layers of the network is increasing, as is the error rate. A residual framework^17^ was proposed to solve this problem, with good results. Different from the one- or two- detector, the main idea of FoveaBox^18^ as an anchor-free detector is to directly learn possible targets and bounding box coordinates in the image without anchors.

Liu Liu et al. proposed a novel method^19^ to improve the accuracy and robustness of large-scale detection and identification of multiple types of pests. This method uses Global activated Feature Pyramid Network (GaFPN) to extract features and Local activated Region Proposal Network (LaRPN) to locate pests. The method they proposed has a great performance in industrial circumstances. Fangyuan Wang et al. proposed a two-stages mobile vision based cascading pest detection approach^20^ to solve the problem of small target detection and data imbalance. Edson Bollis et al. devised a method to automatically select areas of interest to reduce annotations in pest images^21^. Yong He et al. proposed a method to detect oilseed rape pests^22^, which is based on deep learning and can run on mobile platforms. Wang Dawei et al. proposed a diagnostic system based on transfer learning for pest detection and recognition^23^ that a method different from traditional neural networks.Wang Dawei et al. proposed a diagnostic system based on transfer learning for pest detection and recognition, a method different from traditional neural networks. This method exceeds 90% in recognition accuracy. Due to the aphid’s small size, to directly use one or two stages will not have high accuracy. To deal with this issue, detection was divided into two phases^24^. The first stage uses CNNs to find aphid regions, and the second stage uses CNNs to detect insects in aphid regions.

**Figure 1:**
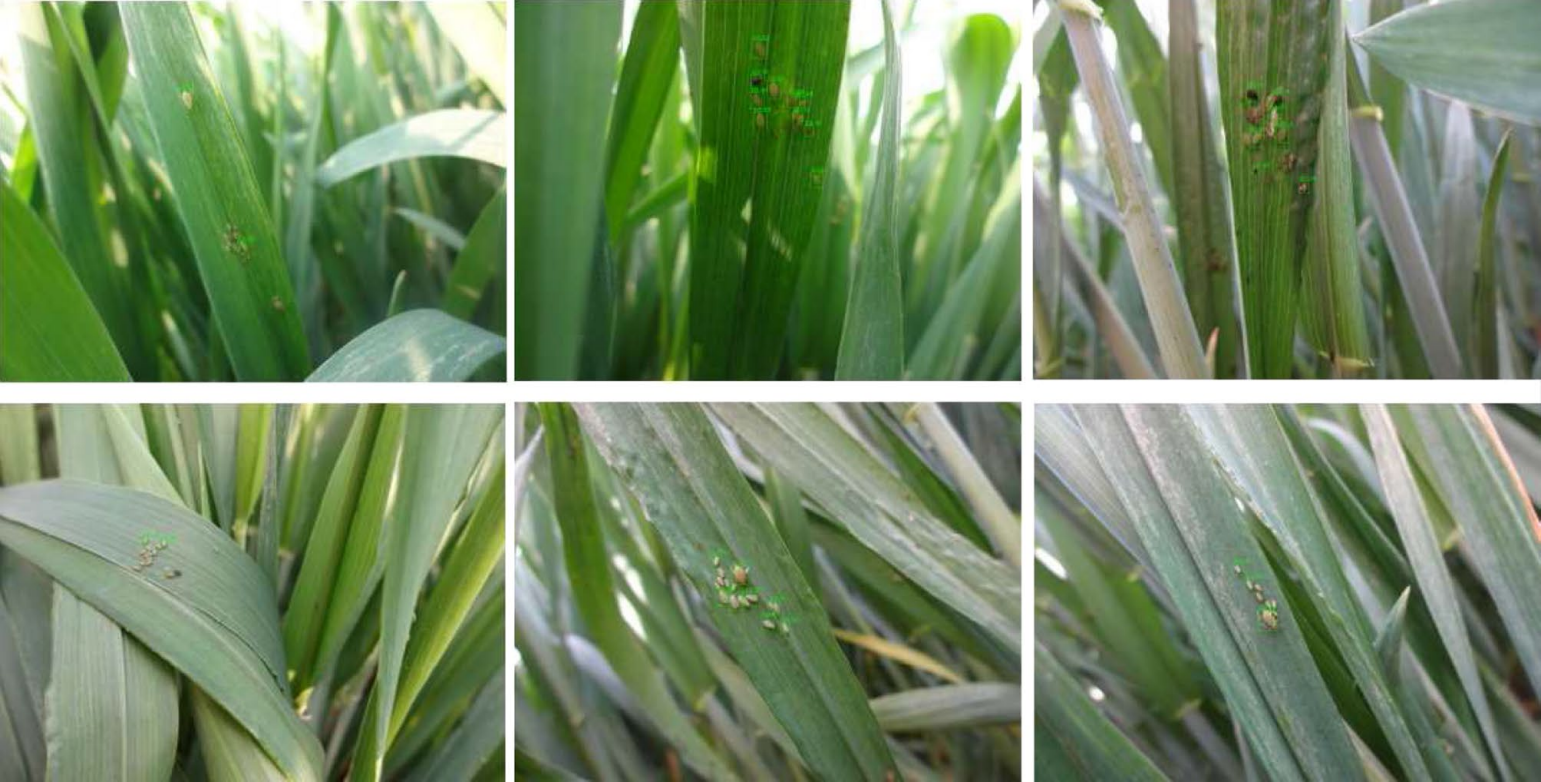
The aphids images are directly fed into the convolutional neural networks. Many aphids are not well detected.

In fact, aphids are easily overlooked in the picture, because their size is too small for the image. It is not just manual observation, but deep learning can hardly detect it well. Specifically, some of the current mainstream detection methods that are directly applied to aphids images may not achieve the expected results in the detection of aphids. As Fig. 1 shows, only a few aphids are found. Even aphids are not detected. The specific reason is that it is difficult to obtain the characteristics of aphids when training the model. In addition, the complex background can weaken the feature extraction, which is also a problem worth considering. In view of the above problems, we propose a novel framework which can improve the accuracy of aphids detection. In order to weaken the influence of complex background on detection, we detected Gaussian blurred images in HSV color space instead of BGR. In order to deal with another problem. a key idea of our proposed algorithm is to treat aphids as special points. we use our proposed method to determine relevant regions based on points that generated by corner points algorithms, which can greatly enhance the features of aphids. In other words, the area where the aphid is located is relatively enlarged. It is better to use convolutional neural networks on sub-regions to train models of features of aphids instead of directly on the whole image.

### Contributions

We propose the enhanced aphid detection framework (EADF) based on ORB and CNN, and use the improved fusion of area of key point algorithm to obtain candidate regions. Finally, a CNN is used for recognition.

The contributions of EADF are summarized as follows:

1. Corner detection is applied to detect aphid areas.
2. HSV and Gaussian blur are used to effectively reduce noise.
3. A novel candidate region algorithm is proposed.
4. The effective association of key points algorithm is used.
5. Coordinate synchronization technology is used.

## Related work

### ORB

Proposed in 2011, ORB^25^ is a feature point detection and description algorithm based on visual information. ORB feature extraction has two parts. (1) Feature point detection uses the FAST^26^ corner detector, which is particularly fast, and direction information is added. (2) Feature point description uses the BRIEF feature descriptor based on comparison of the pixel binary, which improves on the BRIEF^27^ descriptor, which is sensitive to image noise and has no rotation invariance. In the algorithm, we select a point *p* (the center of a circle) with a value of $I_{p}$ and confirm a circle with a radius of 3 units. In this algorithm, 16 pixels ( $I_{pi},i=1,2,...,16$) (with the value of $I_{pi}$) are on the circumference. We select N contiguous pixels from the circumference. Consequence, verify corners by calculating the absolute value of the difference between $p_{i}$ and p became much easier. This process can be described mathematically as

1 $$\begin{aligned} \left\{ \begin{array}{ll} \vert I_{p}-I_{pi} \vert < I_{p}-T\\ \vert I_{p}-I_{pi} \vert > I_{p}+T\\ \end{array} \right. \end{aligned}$$

where T is a threshold.

To expedite calculation, we calculate the absolute values of the successive differences between $p_{i} (i=1,5,9,13)$ and p. If all these results satisfy formula (Eq. 1), then p is a suspected point, and many suspected points are probably contiguous. Nevertheless, we must select the most suitable one. We use the score function,

2 $$\begin{aligned} V=\sum _{i=1}^N \vert I_{p}-I_{pi} \vert \end{aligned}$$

to screen for the point with the highest score.

By a sequence of operations, a large number of points is screened. Then Rublee directly applies the Harris corner measure^28^ to unceasingly filter points. After the filtration process, an intensity centroid^29^ is used to provide corner orientation for filtered points. While the geometric moments have been utilized to calculate the direction of corners. These geometric moments can be defined as

3 $$\begin{aligned} m_{pq}=\sum _{x,y} x^{p}y^{q}I(x,y) \end{aligned}$$

and the geometric centroid can be formulated as:

4 $$\begin{aligned} c=(m_{10}/m_{00},m_{01}/m_{00}) \end{aligned}$$

The angle formed by the line where p and centroid are located with the X-axis is the direction of the key point. The orientation of the patch is defined as

5 $$\begin{aligned} \theta = atan2(m_{01},m_{10}) \end{aligned}$$

The BRIEF algorithm selects n pairs of pixel points $p_i$, $q_i (i = 1,2, ..., n)$ in the neighborhood of each feature point, and compares the magnitude of the gray value of each pair of points. However, as the rotation angle increases, the matching effect of the BRIEF algorithm decreases rapidly. If points selected by BRIEF are to have rotation invariance, they must be multiplied by a rotation matrix. Now, let us assume that P is represented as a neighborhood space of the current corner. Then the binary test $\tau$ can be defined as

6 $$\begin{aligned} \tau (p;x,y):= \left\{ \begin{array}{ll} 1 &{} p(x)<p(y)\\ 0 &{} p(x)\ge p(y)\\ \end{array} \right. \end{aligned}$$

where p(x) is the intensity of p at a point x. A vector of binary tests is defined as:

7 $$\begin{aligned} f_{n}(p):=\sum _{1<i<n} {2^{i-1}-\tau (p;x,y)} \end{aligned}$$

Then a 2 $*$ n matrix will be defined as:

8 $$\begin{aligned} \mathbf {S} = \left( \begin{array}{ccc} x_{1} &{} \ldots &{} x_{n} \\ y_{1} &{} \ldots &{} y_{n} \\ \end{array} \right) \end{aligned}$$

Consequently, the final formula becomes:

9 $$\begin{aligned} S_{\theta } = \mathbf {S}R_{\theta } \end{aligned}$$

10 $$\begin{aligned} g_{n}(p,\theta ):= f_{n}(p)\vee (x_{i},y_{i})\in S_{\theta } \end{aligned}$$

where $R_{\theta }$ is a rotation matrix.

### Convolutional neural network

**Figure 2:**
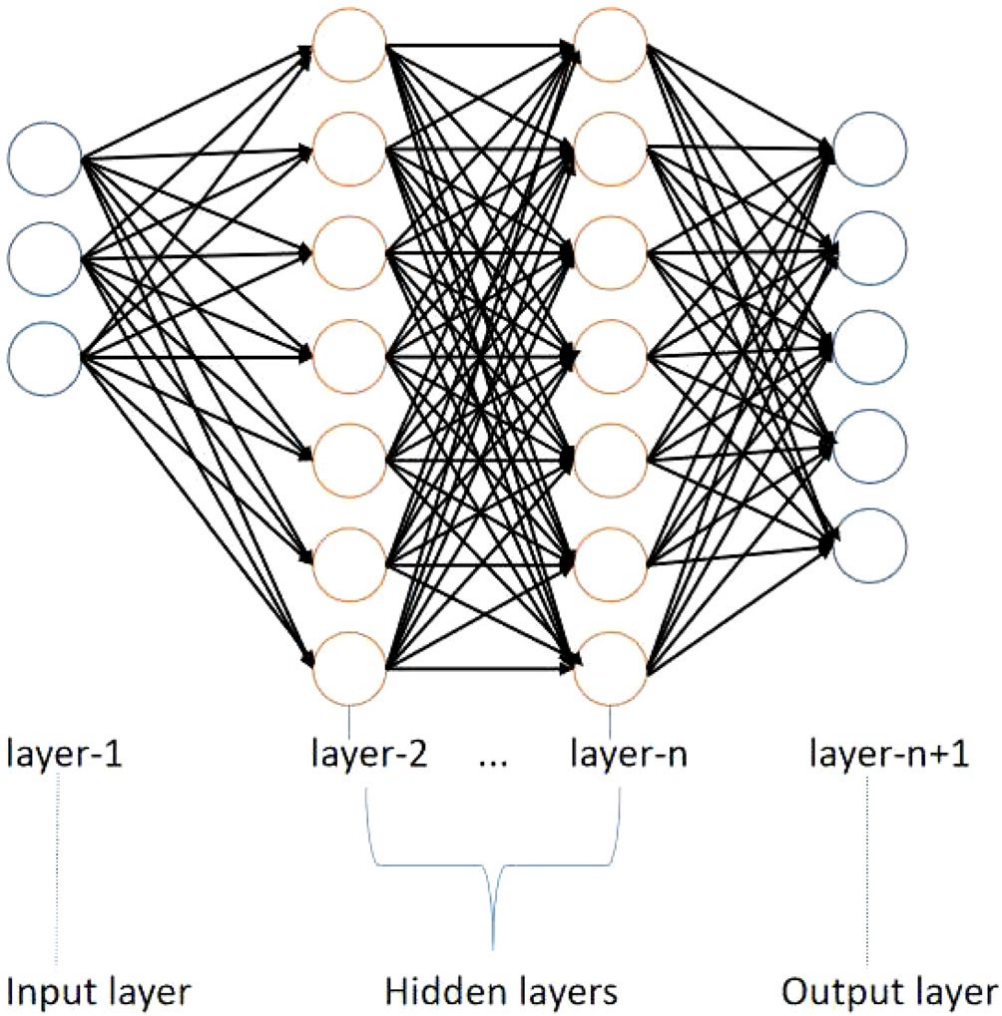
Framework of the neural network.

CNNs are feedforward neural networks with deep learning and deep frameworks, and they have no additional feature engineering requirements for the data. For that reason, a CNN is used in the detection phase as the main method. As shown in Fig. 2, neural network framework consists of an input layer, hidden layer, and output layer.

Most traditional CNNs include convolutional, nonlinear, pooling, and fully connected layers. The convolutional layer is usually the first layer. Some convolutional layers include convolution kernels, which are called local receptive fields or filters. Each kernel represents a feature. After sending a picture to the convolutional layer, these kernels perform convolution operations on it. The closer the features of the kernel and part pictures are, the larger the result of the convolution calculation will be.

The nonlinear layer nonlinearly maps the output of the convolutional layer. Without it, the final input and output will be linear. Common nonlinear functions include sigmoid, tanh, and the rectified linear unit (ReLU). The latter is usually used to attain faster convergence and more quickly obtain gradients.

ReLU is defined as

11 $$\begin{aligned} ReLU(x)= \left\{ \begin{array}{ll} x &{} x>0\\ 0 &{} x<0\\ \end{array} \right. \end{aligned}$$.

Unlike the sigmoid function, it can eliminate the gradient saturation effect. The sigmoid and tanh functions are more common in fully connected layers. Pooling layers are used to make features more prominent. To increase the calculation speed, a pooling layer exploits the features in the previous layer to reduce the redundant data output.

**Figure 3:**
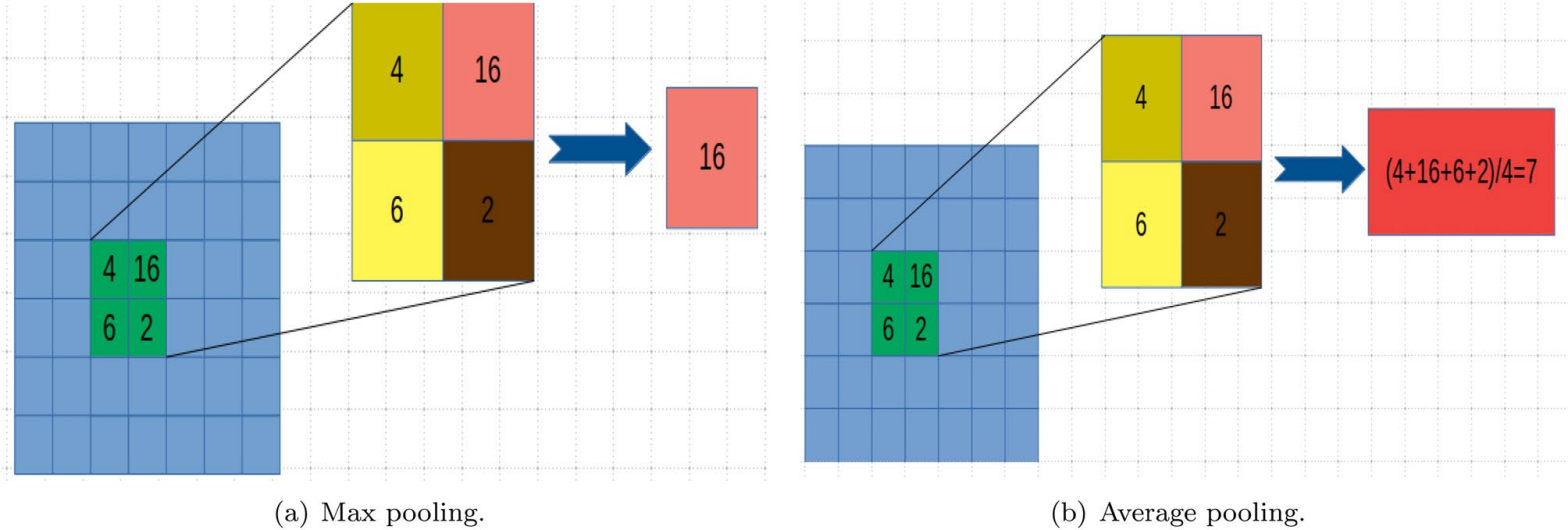
Different pooling methods.

It is worth noting that the pooling operation may lead to the loss of a part of the characteristic data. Max pooling is somewhat more common than average pooling. In the relatively sparse features, maxpooling works better than averagepooling^30^. However, ResNet^17^ and GoogLeNet^31^ use global average pooling. As shown in Fig. 3, the idea of max pooling is to select the largest pixel value in the pooling area, while average pooling takes the average of all values. The fully connected layer actually acts as a classifier. Convolution operations can also replace fully connected layers.

Frankly, based on the powerful feature extraction capabilities of the convolutional neural network, we use it to extract the feature model of aphids based on our proposed method in the “Methods” and utilize this model for detection.

## Methods

### Image acquisition and annotation

**Figure 4:**
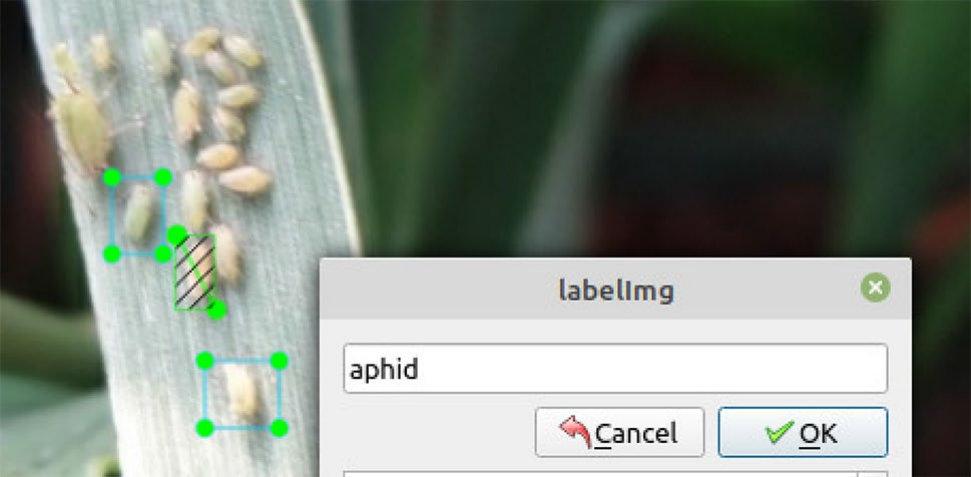
Aphids are annotated and attached to the category.

These pictures of aphids in different numbers and distributions were taken with a focal length of 4 mm and an aperture of f/3.3. Most of them have 1440*1080 pixels. We carefully selected 361 aphids pictures for the data set, of which 207 images are used as the test set and the others as the train set. Labeling is a graphical image annotation tool in Python and Qt that was used to label aphid images. The object was to manually annotate all aphids with rectangular boxes and class names. Aphids were selected sequentially and annotated with a class name, as shown in Fig. 4. Annotations were saved as XML files in PASCAL VOC style, and we used a tool to convert this to the desired CoCo style.

### Technique to enhance aphid detection framework

**Figure 5:**
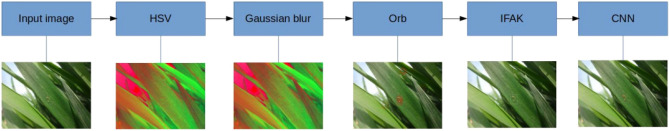
Overall framework of EADF.

We propose to enhance the aphid detection framework based on ORB and CNN, as shown in Fig. 5.

**Figure 6:**
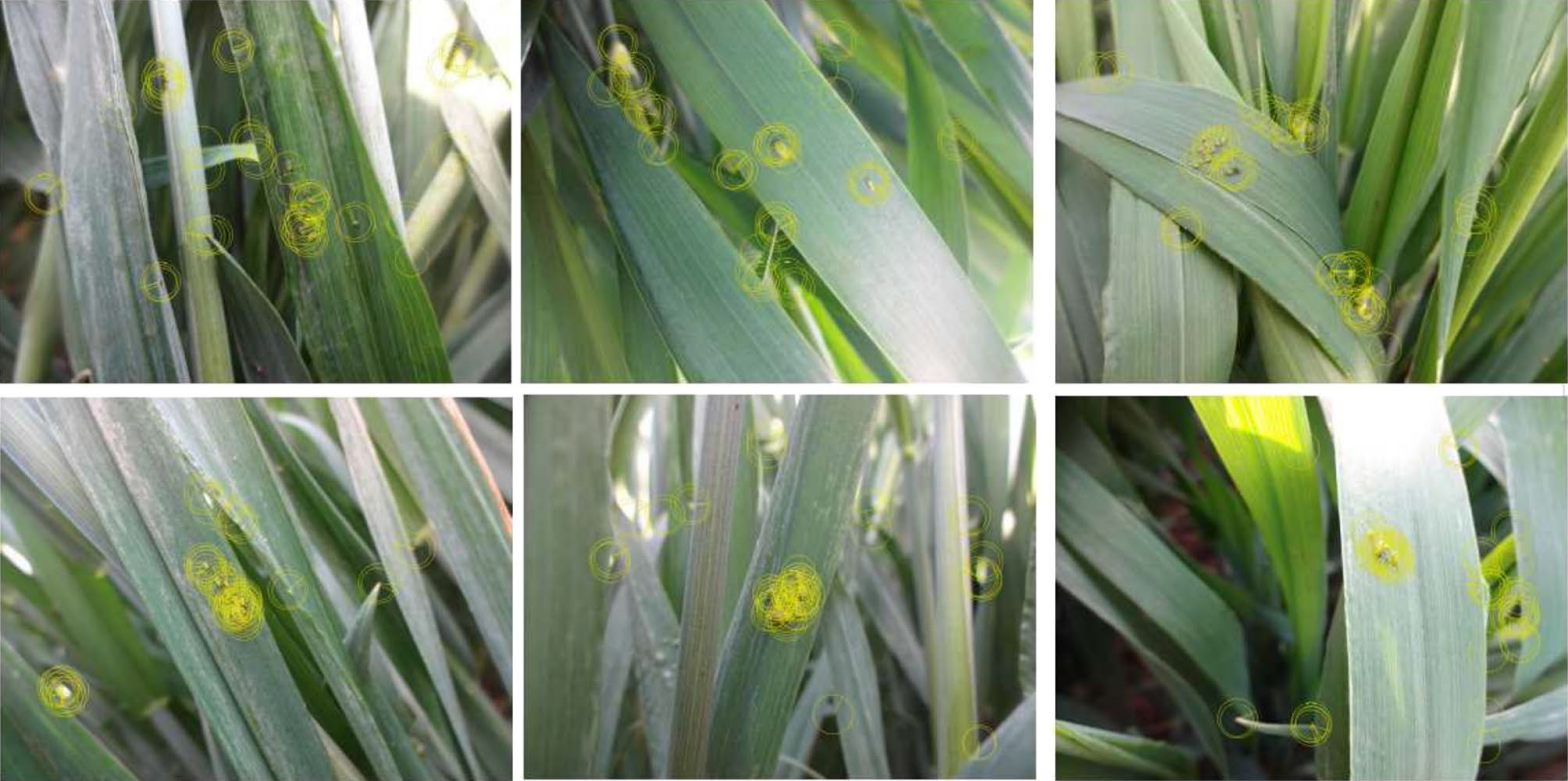
Clique regions are discovered by ORB.

An image must be converted to HSV after being fed into our model. We perform a Gaussian blur for higher noise reduction. Below, we use the experimental results to explain why HSV and Gaussian blur are used for preprocessing. The ORB^25^ algorithm is used on preprocessed images to detect aphid regions. ORB can find the location of the aphid’s area almost exactly, but may generate some extra locations. It can accurately and efficiently replace traditional convolution to determine candidate regions under appropriate conditions. Enormous circles will appear on the image after ORB is applied. These incorporate information such as coordinates of key points, the radius of the circle, and layers of the pyramid. As shown in Fig. 6, clique regions are typically covered by these circles.

The covered regions probably appear to ramble. We propose improved fusion of area of key point (IFAK) to deal with the generated candidate areas. In IFAK, we label the aphids in the candidate region and use a one-stage convolution to train the model. The whole picture is finally split into a 6*6 sub-image. The trained model is applied directly to these sub-images, and all results are integrated.

#### Improved fusion of area of key point (IFAK)

The IFAK algorithm has two parts. First is association of key points, by which several corner regions can be obtained from pictures processed by the ORB algorithm. Some of these areas are singular, and some are clusters. We manually and carefully classify these areas. First, we define a function S(x,y) to obtain the area ratio between the areas formed by two key points. Then we determine two thresholds, t1 and t2, to classify the key points. The classification method is:

12 $$\begin{aligned} t_{1}<S(K_{1},K_{2})<t_{2} \end{aligned}$$

If the relationship between two key points satisfies formula (Eq. 12), then they belong to the same category. When a new key point appears, we need to check whether it belongs to the same category as detected points. Algorithm 1 shows this process.

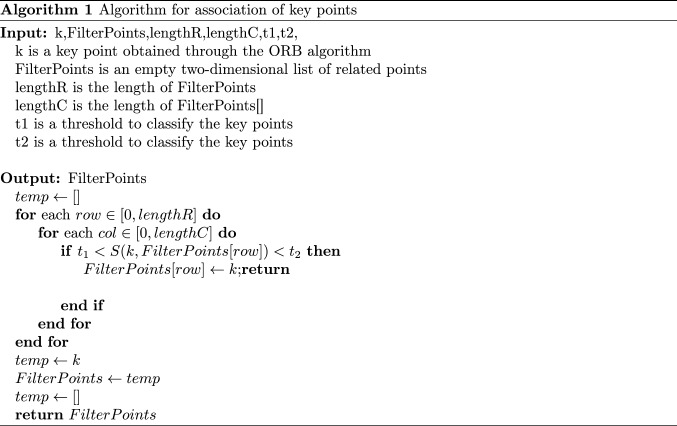



**Figure 7:**
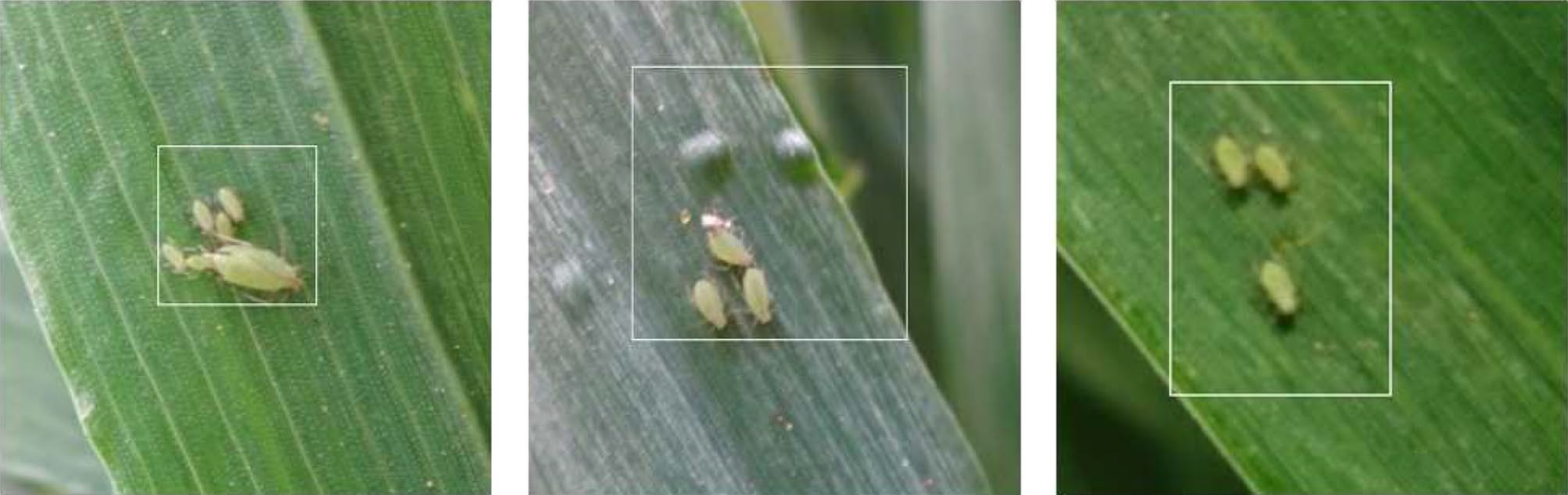
Aphid regions are selected by these boxes produced by the proposed algorithm.

According to the first part, we can smoothly get the key points that have been classified. However, the goal of the second part is to fuse key points of the same class and generate boxes to select aphid regions. Therefore, we use a simple and effective method to generate rectangular boxes. To get the position of the rectangle border, we need to calculate the equation of the line where the four sides of the rectangle are located. The boundary of the minimum frame is obtained through iteration by the center coordinates and radius of the circles that are grouped by Algorithm 1. Each iteration continues to propagate outward until the boundary of the outermost circle is found. This process is shown as Algorithm 2, from whose results we can determine the position of the rectangular box, as shown in Fig. 7.

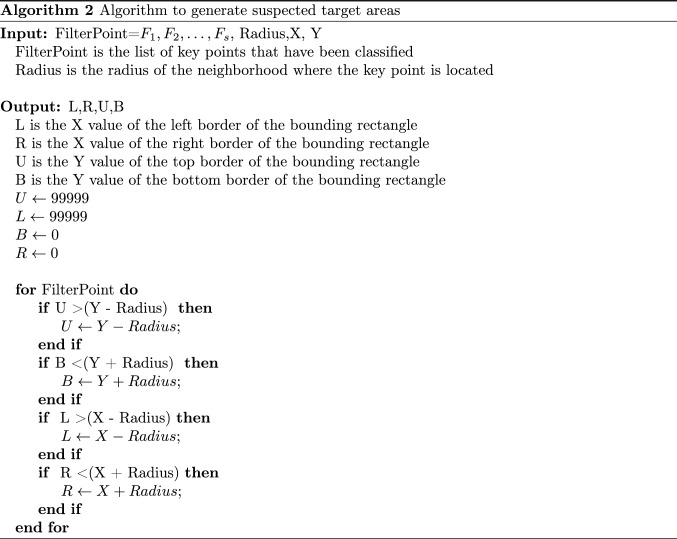



**Figure 8:**
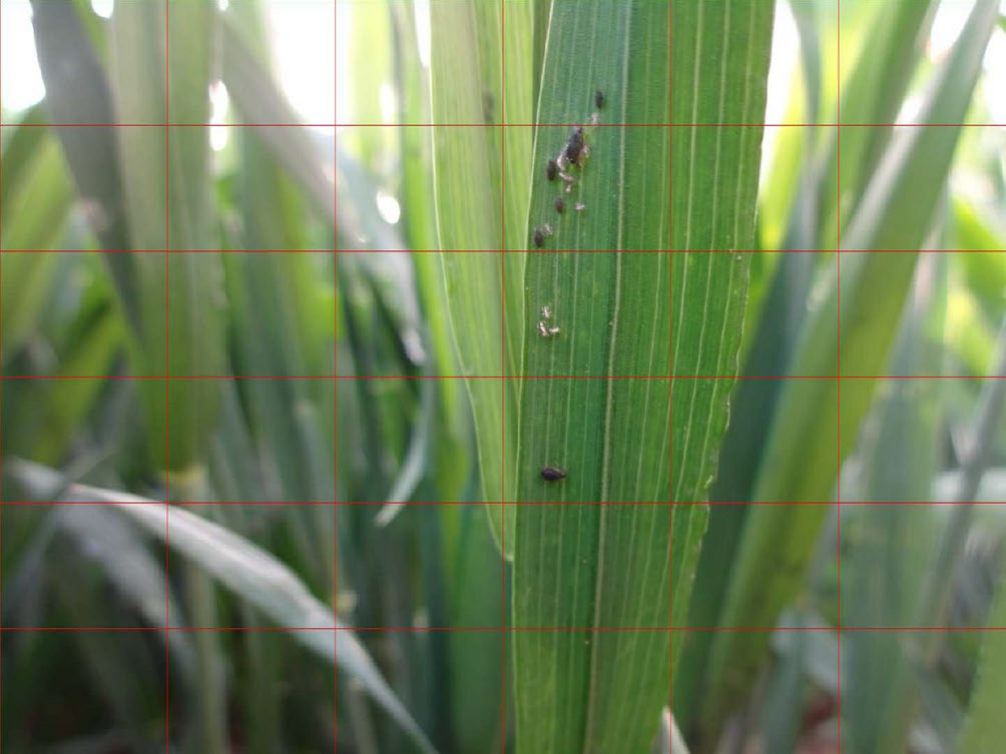
The picture is divided into 36 blocks.

**Figure 9:**
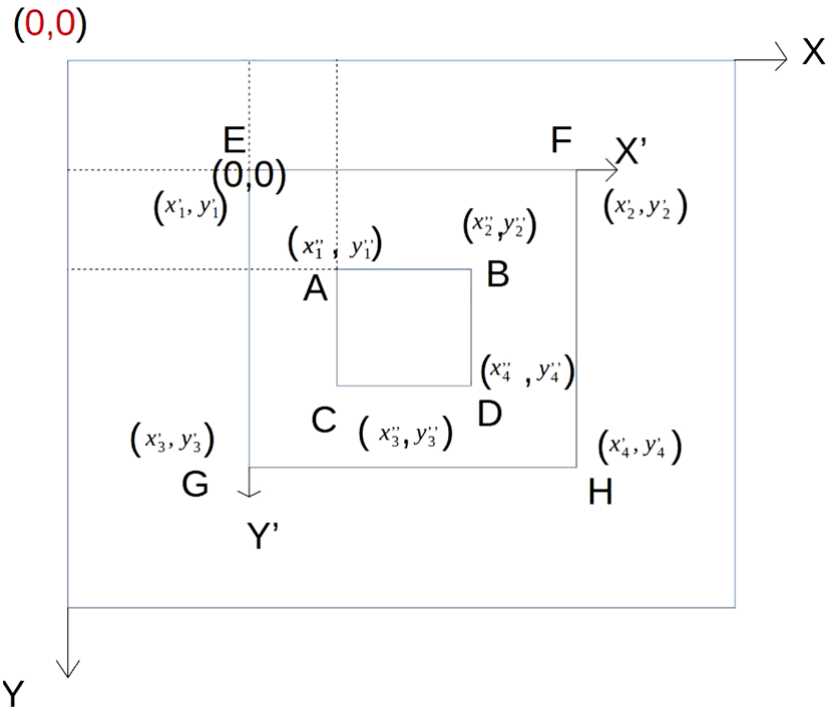
Synchronization of coordinate.

#### Mapping of coordinates

It is necessary to detect pictures containing pests. To better detect targets, we divide the image to be detected into 6*6. Certainly, we can choose how many blocks a picture is divided into. There is no doubt that this is a hyperparameter. Subsequently, a coordinate method of synchronization is well used by us. If we feed a subgraph directly into the CNN, the obtained coordinates are based on this subgraph instead of the whole picture, as shown in Fig. 8. Each sub-picture has independent coordinates, which is not conducive to testing the dataset. Therefore, we map the coordinates of the sub-picture to the whole picture, which can be directly sent to the frame for testing. As shown in Fig. 9, X and Y represent the coordinate system of the whole picture. $X^{'}$ and $Y^{'}$ represent the coordinate system of the subgraph. A, B, C, and D, which are based on the $X^{'}$ and $Y^{'}$ coordinate system, are the corners of the box to which detection targets belong. E, F, G, and H represent the four points of the subgraph, and they use coordinate systems based on both X and Y and $X^{'}$ and $Y^{'}$. We synchronize the coordinates as follows:

13 $$\begin{aligned} \left\{ \begin{array}{ll} A = (x^{'}_{1}+x^{''}_{1},y^{'}_{1}+y^{''}_{1})\\ B = (x^{'}_{1}+x^{''}_{2},y^{'}_{1}+y^{''}_{2})\\ C = (x^{'}_{1}+x^{''}_{3},y^{'}_{1}+y^{''}_{3})\\ D = (x^{'}_{1}+x^{''}_{4},y^{'}_{1}+y^{''}_{4})\\ \end{array} \right. \end{aligned}$$

### Evaluation metrics

In terms of evaluation metrics, average precision(AP)^32^ and average recall(AR)^33^ are selected as metrics in order to verify the performance of our model on aphids detection. Before calculating AP and AR, True Positive(TP), False Positive(FP) and False Negative(FN) samples need to be determined. The Precision-Recall (PR) is calculated by:

14 $$\begin{aligned} \left\{ \begin{array}{ll} precision=\dfrac{TP}{TP+FP}\\ recall=\dfrac{TP}{TP+FN}\\ \end{array} \right. \end{aligned}$$

the AP is defined as the area under Precision-Recall with an Intersection over Union(IoU) threshold. Therefore, AP can be expressed by calculus.

15 $$\begin{aligned} AP=\int precision \ d(recall) \end{aligned}$$

Average recall (AR) between 0.5 and 1 can be obtained by formula 16.

16 $$\begin{aligned} AR=2*\int _{0.5}^{1}recall(x)dx \end{aligned}$$

## Experiment and discussion

### Experimental settings and environment

In this article, PyCharm is the platform for executing our code and contrast the code. The proposed structure runs on Linux with a 6G GTX1660Ti GPU and an Intel i5-9300H CPU, programmed in Python 3.6, with OpenCV-Python 4.1.2 and MMDetection^34^. The learning rate, momentum and weight decay are initialized to 0.01, 0.9 and 0.001 respectively. In addition, the gradient update rule uses Stochastic Gradient Descent (SGD). A variety of CNNs structures are selected to extract the features of aphids.

### Preprocessing analysis and results

**Figure 10:**
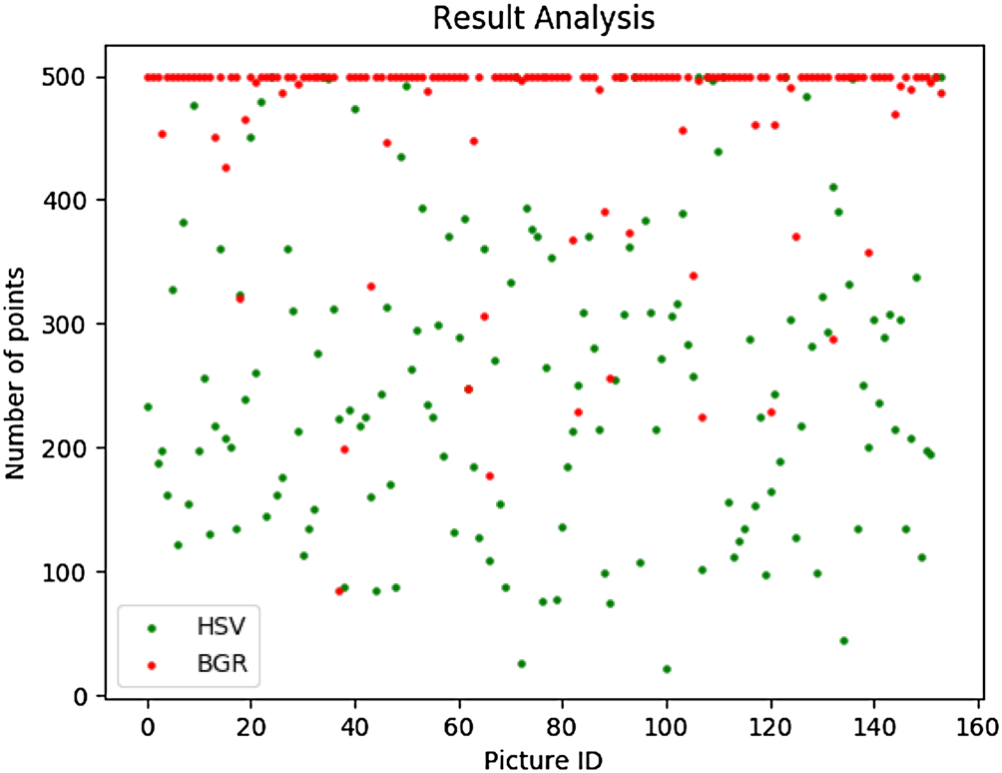
Comparison of HSV and BGR.

**Figure 11:**
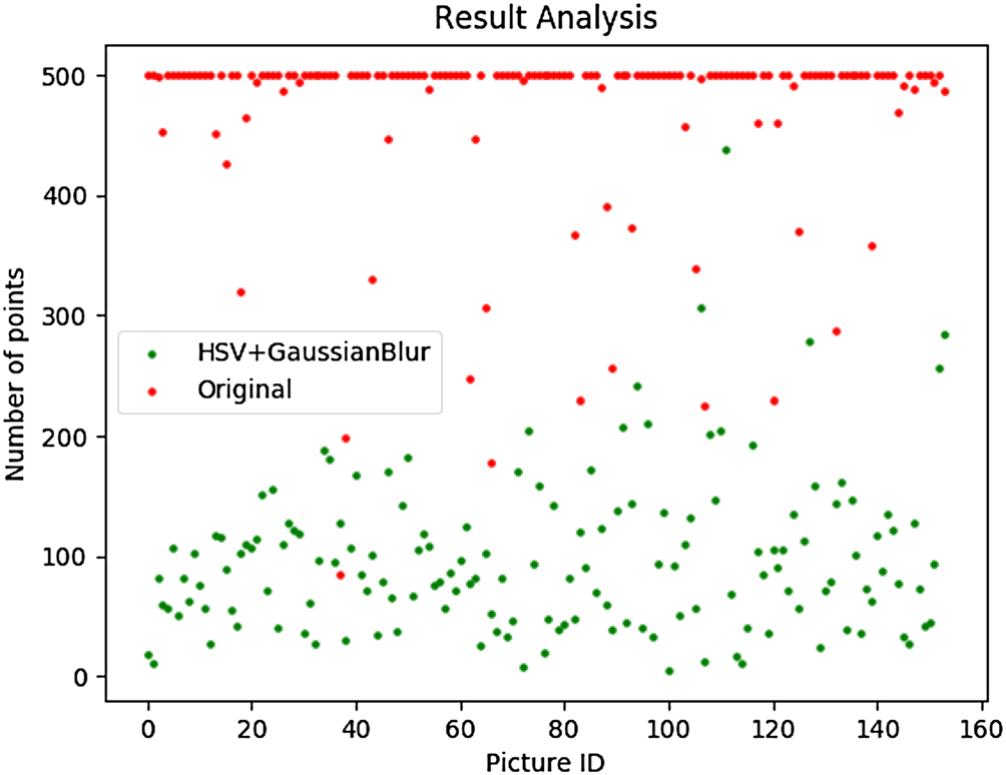
Comparison of image converted to HSV by Gaussian blur and original image.

The input picture must be preprocessed before executing the proposed algorithm. OpenCV loads images in BGR format; hence pictures must be converted to HSV, which can produce fewer redundant points. The aphid image in Fig. 10 performs better in HSV format. The converted image must then be processed by Gaussian blur. Experimental results show that the converted image can remove many points, except those at the borders of leaves cannot be removed well. Gaussian blur is used on the converted image to eliminate the extra points. Fig. 11 shows the results of the comparison.

###  Candidate region analysis and results

We check the results of the IFAK algorithm under different optimization conditions. Table 1 shows the average number of candidate regions in each image under different conditions. It is obvious that the proposed method significantly reduces the number of candidate regions.

**Table 1:** Average number of candidate regions by method, where key points are selected from the sixth and seventh layers in the pyramid.

Models	Average number of candidate regions
Original $+$ IFAK	8.909
HSV $+$ IFAK	7.487
Gaussian blur $+$ IFAK	8.902
HSV $+$ Gaussian blur $+$ IFAK	6.759

### Results and analysis of aphids detection

Four one-stage methods with different backbone networks were used for comparative experiments. RetinaNet-EADF,SSD-EADF are the approaches using EADF,the others are methods without EADF. Bold font is used to indicate the best results. The experimental results of one-stage methods are shown in Table 2, which demonstrates that one-stage methods using EADF have better AP and AR. The experiment shows that EADF can improve results. The AP value of RetinaNet with EADF using ResNet-50 as backbone is 0.385 higher than that of RetinaNet. Another RetinaNet that uses deeper layers gets better AP values. Both RetinaNet and SSD achieve better AP and AR values if they use EADF.

**Table 2:** Comparison of detection results based on one-stage with and without EADF.

Models	Backbone	AP	$AP_{50}$	$AP_{75}$	$AP_{S}$	$AP_{M}$	$AR_{1}$	$AR_{10}$	$AR_{100}$	$AR_{S}$	$AR_{M}$
**RetinaNet-EADF**	ResNet-50	0.439	0.797	0.452	0.428	0.474	0.047	0.343	0.515	0.507	0.535
RetinaNet	ResNet-50	0.054	0.214	0.006	0.017	0.152	0.017	0.072	0.201	0.153	0.336
**RetinaNet-EADF**	ResNet-101	0.442	0.792	0.475	0.433	0.471	0.047	0.343	0.521	0.516	0.536
RetinaNet	ResNet-101	0.117	0.425	0.016	0.067	0.235	0.023	0.132	0.260	0.214	0.391
**SSD-EADF**	SSD300-VGG16	0.313	0.624	0.286	0.297	0.359	0.041	0.282	0.429	0.421	0.452
SSD	SSD300-VGG16	0.027	0.121	0.003	0.008	0.079	0.012	0.059	0.116	0.078	0.223
**SSD-EADF**	SSD512-VGG16	0.348	0.651	0.342	0.339	0.374	0.044	0.297	0.463	0.457	0.481
SSD	SSD512-VGG16	0.181	0.559	0.056	0.136	0.301	0.031	0.189	0.290	0.244	0.423

Then, we conduct experiments on the two-stage method and the anchor-free method. Table 3 records the experimental results of FasterRCNN, CascadeRCNN and FoveaBox.We can find that two-stage methods using EADF have better AP and AR,as well as the anchor-free method, which is similar to the results in Table 2. In the following the best results, the difference between the method with EADF and the method without it is 0.281 in the AP. Certainly, the AR value has also been improved. In the worst results of the experiment, we still can find that the method with EADF is more than without it by 43.3% on value of AP.

**Table 3:** Comparison of detection results based on two-stage and anchor-free with and without EADF.

Models	Backbone	AP	$AP_{50}$	$AP_{75}$	$AP_{S}$	$AP_{M}$	$AR_{1}$	$AR_{10}$	$AR_{100}$	$AR_{S}$	$AR_{M}$
FasterRCNN	ResNet-50	0.265	0.724	0.110	0.240	0.338	0.030	0.246	0.367	0.325	0.487
**FasterRCNN-EADF**	ResNet-50	0.463	0.817	0.494	0.445	0.504	0.049	0.355	0.543	0.534	0.566
FasterRCNN	ResNet-101	0.305	0.760	0.166	0.295	0.355	0.032	0.271	0.402	0.369	0.495
**FasterRCNN-EADF**	ResNet-101	0.472	0.821	0.511	0.458	0.513	0.048	0.364	0.554	0.545	0.578
CascadeRCNN	ResNet-50	0.294	0.753	0.144	0.267	0.375	0.032	0.261	0.396	0.352	0.522
**CascadeRCNN-EADF**	ResNet-50	0.473	0.812	0.523	0.457	0.514	0.048	0.361	0.543	0.534	0.569
CascadeRCNN	ResNet-101	0.330	0.789	0.196	0.321	0.381	0.035	0.282	0.428	0.397	0.514
**CascadeRCNN-EADF**	ResNet-101	0.473	0.798	0.525	0.460	0.509	0.050	0.362	0.546	0.538	0.568
FoveaBox	ResNet-50	0.146	0.545	0.018	0.123	0.226	0.022	0.162	0.257	0.212	0.384
**FoveaBox-EADF**	ResNet-50	0.427	0.767	0.444	0.417	0.452	0.046	0.337	0.516	0.511	0.530

In addition, we also compared the two-stage method without EADF with other methods with EADF. Table 4 shows that the two-stage method without EADF neither AP nor AR can reach the value of the method with EADF. In other words, our proposed method can improve the accuracy of aphids detection. The best detection result for two-stage detectors is close to the worst detection result for one-stage detectors, i.e., 0.330 vs. 0.313. It is worth noting that the best result for RetinaNet is 60 percent more than Faster-RCNN in AP.

**Table 4:** Comparison of other methods with EADF and two-stage without EADF.

Model	Backbone	AP	$AP_{50}$	$AP_{75}$	$AP_{S}$	$AP_{M}$	$AR_{1}$	$AR_{10}$	$AR_{100}$	$AR_{S}$	$AR_{M}$
**RetinaNet-EADF**	ResNet-50	0.439	0.797	0.452	0.428	0.474	0.047	0.343	0.515	0.507	0.535
FasterRCNN	ResNet-50	0.265	0.724	0.110	0.240	0.338	0.030	0.246	0.367	0.325	0.487
**RetinaNet-EADF**	ResNet-101	0.442	0.792	0.475	0.433	0.471	0.047	0.343	0.521	0.516	0.536
FasterRCNN	ResNet-101	0.305	0.760	0.166	0.295	0.355	0.032	0.271	0.402	0.369	0.495
**SSD-EADF**	SSD512-VGG16	0.348	0.651	0.342	0.339	0.374	0.044	0.297	0.463	0.457	0.481
CascadeRCNN	ResNet-50	0.294	0.753	0.144	0.267	0.375	0.032	0.261	0.396	0.352	0.522
CascadeRCNN	ResNet-101	0.330	0.789	0.196	0.321	0.381	0.035	0.282	0.428	0.397	0.514
**SSD-EADF**	SSD300-VGG16	0.313	0.624	0.286	0.297	0.359	0.041	0.282	0.429	0.421	0.452
**FoveaBox-EADF**	ResNet-50	0.427	0.767	0.444	0.417	0.452	0.046	0.337	0.516	0.511	0.530

**Figure 12:**
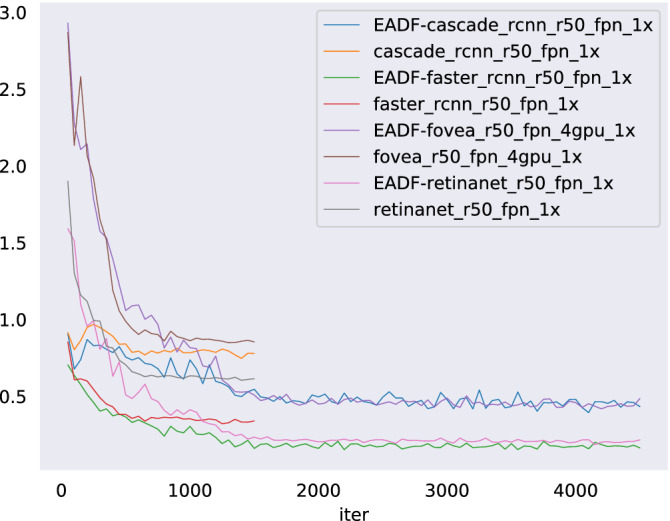
The loss value obtained by different detection methods with 50 layers during training.

**Figure 13:**
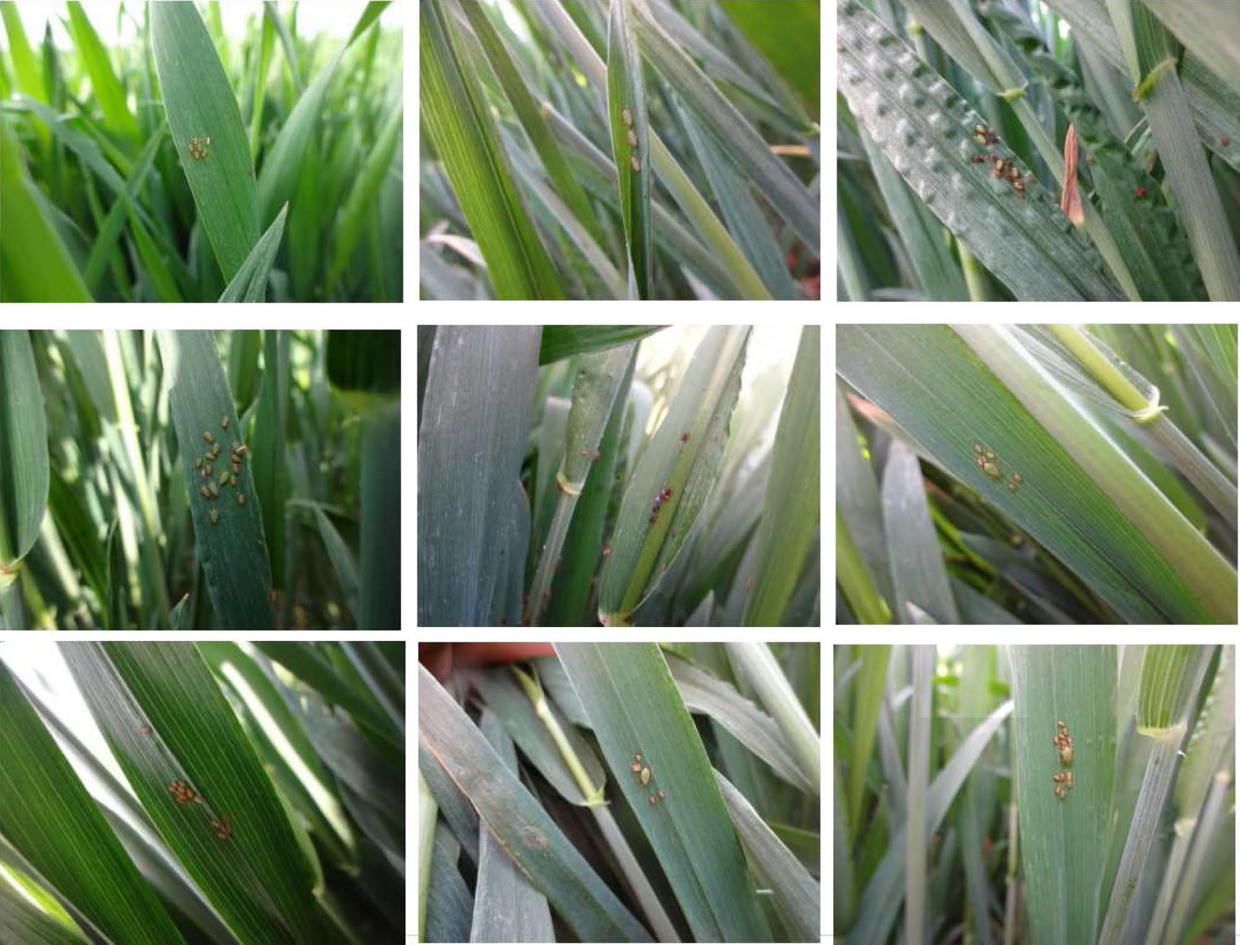
The results of using EADS to detect aphids.

From the results in Tables 2, 3 and 4, we can easily find a clear conclusion that the aphids detection framework proposed by us is able to greatly improve the average accuracy and average recall of aphids detection by general detectors. Figure 12 clearly proves the above point. The methods with our proposed framework obviously has a better curve than others during training. Because our method can find suspected aphid regions, which makes the convolutional neural networks directly utilize these areas to train the model instead of the entire image. Specifically, the aphids are amplified, which strengthens the features obtained by convolutional neural networks. Therefore, our method can improve the accuracy of aphids detection. Figure 13 shows the results of aphids detection by our method.

## Conclusion

In this paper, we propose an aphid detection framework called EADS to detect aphids in different distributions under natural conditions. In the proposed method, Images are converted from BGR to HSV, and then they are processed by Gaussian blur to further deal with noise. IFAK is used to generate candidate boxes. Next, the model is trained in the aphid candidate area using CNNs. Then, the trained model is used to detect aphids on the segmented sub-images. Finally, all the detection results are fused through coordinate mapping to obtain a complete aphid detection result.The proposed method was evaluated on several algorithms and compared with a number of recent methods. Experimental results indicate that the proposed system can well improve the detection accuracy of these algorithms. The major contribution of EADS is: improved fusion of key points method is proposed. This method quickly finds the suspected aphid regions to further improve the weight of the aphid feature in the model. In the future, our goal is to improve EADS so that it can be applied to general small target detection tasks.

## References

[1] Dalal, N., & Triggs, B.. Histograms of oriented gradients for human detection. in *2005 IEEE Computer Society Conference on Computer Vision and Pattern Recognition (CVPR’05)*, Vol. 1, 886–893 (2005).

[2] Felzenszwalb PF, Girshick RB, McAllester D, Ramanan D (2010). Object detection with discriminatively trained part-based models. IEEE Trans. Pattern Anal. Mach. Intell..

[3] Uijlings JR, Sande KE, Gevers T, Smeulders AW (2013). Selective search for object recognition. Int. J. Comput. Vis..

[4] Lecun, Y., Bottou, L., Bengio, Y., & Haffner, P. Gradient-based learning applied to document recognition. *Intell. Signal Process.* 306–351 (2001).

[5] Krizhevsky A, Sutskever I, Hinton GE (2012). Imagenet classification with deep convolutional neural networks. Adv. Neural Inf. Process. Syst..

[6] Simonyan, K. & Zisserman, A. Very deep convolutional networks for large-scale image recognition. in *ICLR 2015 : International Conference on Learning Representations 2015* (2015).

[7] Redmon, J., Divvala, S., Girshick, R., & Farhadi, A. You only look once: Unified, real-time object detection. in *2016 IEEE Conference on Computer Vision and Pattern Recognition (CVPR)*, 779–788 (2016).

[8] Liu, W., Anguelov, D., Erhan, Dumitru, S., Christian, R., Scott, E., Fu, C.-Y., & Berg, A.C. Ssd: Single shot multibox detector. in *European Conference on Computer Vision*, 21–37 (2016).

[9] Law H, Deng J (2020). Cornernet: Detecting objects as paired keypoints. Int. J. Comput. Vis..

[10] Lin T-Y, Goyal P, Girshick R, He K, Dollar P (2020). Focal loss for dense object detection. IEEE Trans. Pattern Anal. Mach. Intell..

[11] Girshick, R., Donahue, J., Darrell, T., & Malik, J. Rich feature hierarchies for accurate object detection and semantic segmentation. in *CVPR ’14 Proceedings of the 2014 IEEE Conference on Computer Vision and Pattern Recognition*, 580–587 (2014).

[12] Girshick, R. Fast r-cnn. in *2015 IEEE International Conference on Computer Vision (ICCV)*, 1440–1448 (2015).

[13] Ren S, He K, Girshick R, Sun J (2017). Faster r-cnn: Towards real-time object detection with region proposal networks. IEEE Trans. Pattern Anal. Mach. Intell..

[14] Cai, Z., Vasconcelos, N. Cascade r-cnn: Delving into high quality object detection. in *2018 IEEE/CVF Conference on Computer Vision and Pattern Recognition*, 6154–6162 (2018).

[15] Xue B, Tong N, Xin X (2019). Diod: Fast, semi-supervised deep isar object detection. IEEE Sens. J..

[16] Yan, Q., Yang, B., Wang, W., Wang, B., Chen, P., Zhang, J. Apple leaf diseases recognition based on an improved convolutional neural network. *Sensors* 3535 (2020). 10.3390/s20123535 PMC7349496 32580395

[17] He, K., Zhang, X., Ren, S., & Sun, J. Deep residual learning for image recognition. in *2016 IEEE Conference on Computer Vision and Pattern Recognition (CVPR)*, 770–778 (2016).

[18] Kong T, Sun F, Liu H, Jiang Y, Li L, Shi J (2020). Foveabox: Beyound anchor-based object detection. IEEE Trans. Image Process..

[19] Liu, L., Xie, C., Wang, R., Yang, P., Sudirman, S., Zhang, J., Li, R., & Wang, F. Deep learning based automatic multi-class wild pest monitoring approach using hybrid global and local activated features. *IEEE Trans. Ind. Inform.* 1–1 (2020).

[20] Wang F, Wang R, Xie C, Yang P, Liu L (2020). Fusing multi-scale context-aware information representation for automatic in-field pest detection and recognition. Comput. Electron. Agric..

[21] Bollis, E., Pedrini, H., & Avila, S. Weakly supervised learning guided by activation mapping applied to a novel citrus pest benchmark. in *2020 IEEE/CVF Conference on Computer Vision and Pattern Recognition Workshops (CVPRW)* (2020).

[22] He, Y., Zeng, H., Fan, Y., Ji, S. & Jianjian, W. Application of deep learning in integrated pest management: A real-time system for detection and diagnosis of oilseed rape pests. *Mobile Inf. Syst.* **1–14**, 2019 (2019).

[23] Dawei W, Limiao D, Jiangong N, Jiyue G, Hongfei Z, Zhongzhi H (2019). Recognition pest by image-based transfer learning. J. Sci. Food Agric..

[24] Li R, Wang R, Xie C, Liu L, Zhang J, Wang F, Liu W (2019). A coarse-to-fine network for aphid recognition and detection in the field. Biosyst. Eng..

[25] Rublee, E., Rabaud, V., Konolige, K., & Bradski, G. Orb: An efficient alternative to sift or surf. in *2011 International Conference on Computer Vision*, 2564–2571 (2011).

[26] Rosten, E., & Drummond, T. Machine learning for high-speed corner detection. in *Lecture Notes in Computer Science*, 430–443 (2006).

[27] Calonder, M., Lepetit, V., Strecha, C., & Pascal, F. Brief: Binary robust independent elementary features. in *ECCV’10 Proceedings of the 11th European Conference on Computer Vision: Part IV*, Vol 6314, 778–792 (2010).

[28] Harris, C.G., & Stephens, M. A combined corner and edge detector. in *Proceedings of the 4th Alvey Vision Conference, Manchester, U.K., Aug. 1988*, 147–151 (1988).

[29] Rosin PL (1999). Measuring corner properties. Comput. Vis. Image Underst..

[30] Boureau, Y., Ponce, J., & Lecun, Y. A theoretical analysis of feature pooling in visual recognition. in *Proceedings of the 27th International Conference on Machine Learning*, 111–118 (2010).

[31] Szegedy, C., Liu, W., Jia, Y., Sermanet, P., Reed, S., Anguelov, D., Erhan, D., Vanhoucke, V., & Rabinovich, A. Going deeper with convolutions. in *2015 IEEE Conference on Computer Vision and Pattern Recognition (CVPR)*, 1–9 (2015).

[32] Zhang, E., & Zhang, Y. *Average Precision*, 1–1. (Springer New York, 2016).

[33] Hosang J, Benenson R, Dollar P, Schiele B (2016). What makes for effective detection proposals. IEEE Trans. Pattern Anal. Mach. Intell..

[34] Chen, K., Wang, J., Pang, J., Cao, Y., Xiong, Y., Li, X., Sun, S., Feng, W., Liu, Z., Xu, J., Zhang, Z., Cheng, D., Zhu, C., Cheng, T., Zhao, Q., Li, B., Lu, X., Zhu, R., Wu, Y., Dai, J., Wang, J., Shi, J., Ouyang, W., Change Loy, C., & Lin, D. MMDetection: Open mmlab detection toolbox and benchmark. arXiv preprint arXiv:1906.07155 (2019).

